# Supplementary material for: The interactome of intact mitochondria by cross-linking mass spectrometry provides evidence for coexisting respiratory supercomplexes
Source: Mol Cell Proteomics. 2017 Dec 8;17(2):216–32. doi: 10.1074/mcp.RA117.000470 (PMC5795388; doi:10.1074/mcp.RA117.000470)
Supplement: Supplemental Data [file supp_RA117.000470_133922_0_supp_23978_fzffwf.zip › spectra_annotation/mito_spectra_annotation/400-1-4-1-10-1.pdf]

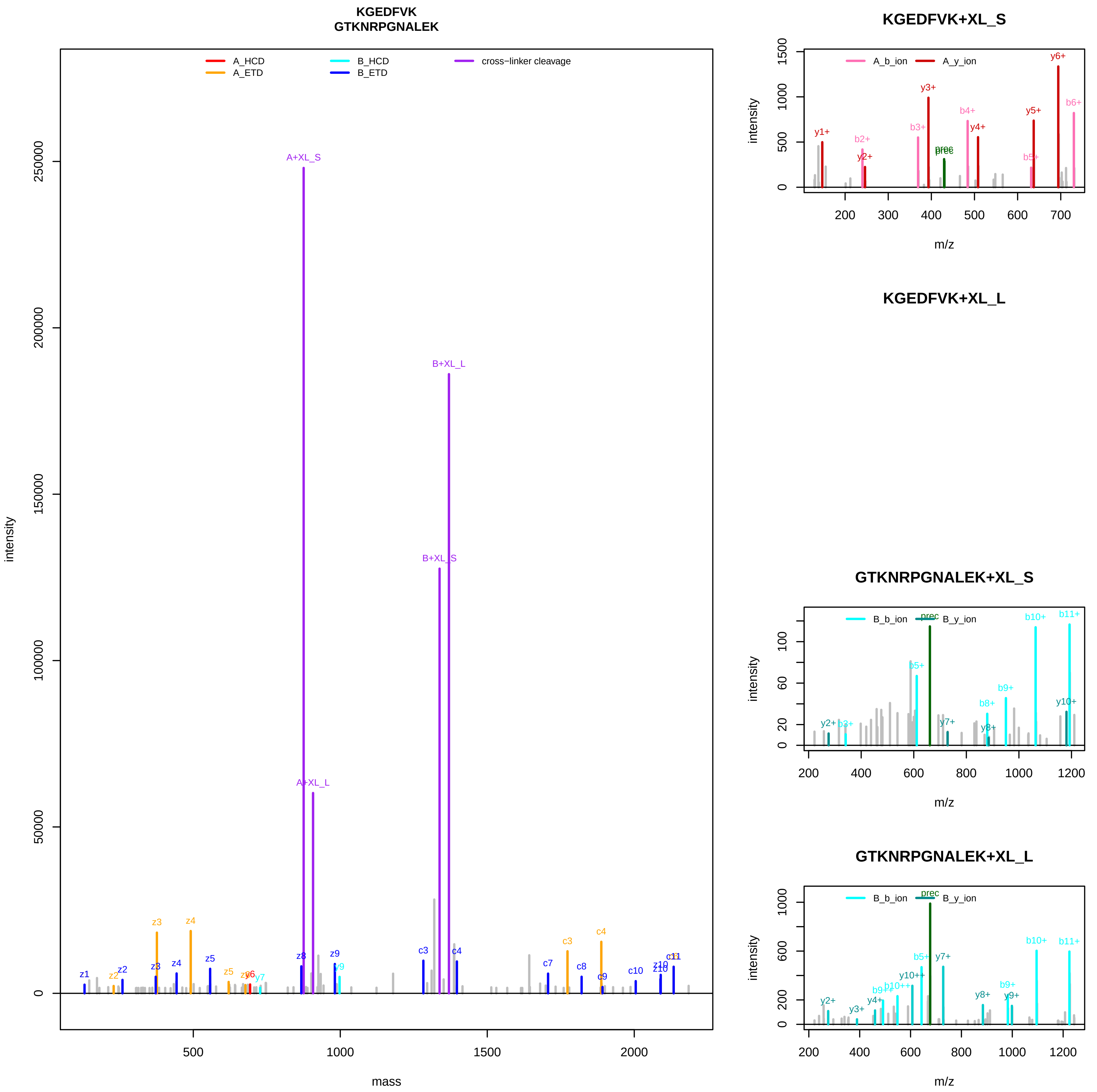
KGEDFVK
GTKNRPGNALEK
A_HCD
A_ETD
B_HCD
B_ETD
cross−linker cleavage
A+XL_S
B+XL_L
B+XL_S
A+XL_L
z1 z2 z3 z4 z5 z8 z9 y7 y9 c3 c4 c7 c8 c9 c10 z10 c11
intensity
mass
0
50000
100000
150000
200000
250000
500
1000
1500
2000
KGEDFVK+XL_S
A_b_ion
A_y_ion
y1+ y2+ y3+ y4+ y5+ y6+ b2+ b3+ b4+ b5+ b6+ prec
m/z
KGEDFVK+XL_L
GTKNRPGNALEK+XL_S
B_b_ion
B_y_ion
y2+ b3+ b5+ y7+ y8+ b8+ b9+ b10+ b11+ y10+ prec
GTKNRPGNALEK+XL_L
y2+ y3+ y4+ b9++ b10++ y10++ b5+ y7+ y8+ b9+ y9+ b10+ b11+ prec
